# Bactericidal Effect of a Novel Phage Endolysin Targeting Multi-Drug-Resistant *Acinetobacter baumannii*

**DOI:** 10.3390/antibiotics14020162

**Published:** 2025-02-06

**Authors:** Sara Garcia Torres, Dirk Henrich, Rene D. Verboket, Ingo Marzi, Gernot Hahne, Volkhard A. J. Kempf, Stephan Göttig

**Affiliations:** 1 Goethe University Frankfurt, University Hospital, Institute of Medical Microbiology and Infection Control, 60596 Frankfurt am Main, Germany; garciatorres@med.uni-frankfurt.de; 2 Goethe University Frankfurt, University Hospital, Department of Trauma Surgery and Orthopedics, 60590 Frankfurt am Main, Germany; d.henrich@trauma.uni-frankfurt.de (D.H.); rene.verboket@ukffm.de (R.D.V.); marzi@trauma.uni-frankfurt.de (I.M.); 3 Lysando Innovations Lab GmbH, 93053 Regensburg, Germany; gernot.hahne@lysando.com

**Keywords:** endolysins, *Acinetobacter baumannii*, antibacterial treatment, activity, resistance

## Abstract

Background/Objectives: Infections with antibiotic-resistant Gram-negative pathogens represent a major global threat to public health. *Acinetobacter baumannii* is a highly important nosocomial pathogen causing severe and life-threatening infections, like pneumonia, wound infections, or sepsis. It is often resistant even against last-resort antibiotics, such as carbapenems, and can persist in healthcare settings. Artilysin^®^s are a novel class of endolysins targeted against multidrug-resistant bacteria. Methods: Antibacterial activity of Art-Top3 was determined by broth microdilution, in vitro assays and in the *Galleria mellonella* infection model. The toxicity of Art-Top3 on red blood cells, endothelial and epithelial cells was analyzed using the MTT assay. Results: Here, we report on a new Artilysin^®^ Art-Top3 that is active against *A. baumannii* and led to a 10^5^-fold reduction in viable *A. baumannii* after five minutes of exposure. Art-Top3 showed activity against *A. baumannii* biofilms in static and dynamic experimental infection models. Furthermore, upon infection with carbapenem-resistant *A. baumannii* patient isolates, Art-Top3 was able to rescue human primary cells in vitro and larvae of *Galleria mellonella* in an in vivo infection model. Art-Top3 did not lyse human red blood cells and showed activity in human serum, indicating a low toxicity and high stability of Art-Top3 in vitro. Conclusion: Our findings collectively establish that Art-Top3 might be a candidate for novel therapeutic strategies of infections caused by multidrug-resistant *A. baumannii* pathogens.

## 1. Introduction

The widespread emergence of antimicrobial infections with multidrug-resistant (MDR) Gram-negative bacteria, such as *Acinetobacter baumannii*, is becoming an increasing public health threat by causing severe diseases and surviving for weeks on the surfaces of hospital facilities and equipment [1]. *A. baumannii* is one of the most important pathogens responsible for hospital-acquired infections, including urinary tract, bloodstream, and wound infections, as well as ventilator-associated pneumonia [1,2]. The mortality rate in intensive care units can be up to 40% [3] and, in some cases with MDR strains, up to 70% [4]. The severity is often higher in critically ill patients, patients with prolonged hospitalization, immunosuppression, severe trauma, or burns, older patients, and ventilated patients [5]. Resistance of *A. baumannii* to several β- lactam antibiotics has been reported, either by intrinsic mechanisms, such as expression OXA-51-like β-lactamases, or through the acquisition of carbapenemases [6]. Moreover, various mechanisms such as efflux pumps, aminoglycoside-modifying enzymes, permeability defects, and modification of target sites facilitate *A. baumannii* resistance to multiple classes of antibiotics [7,8], leading to a high frequency of multidrug-resistant *A. baumannii* strains worldwide. The high genetic plasticity of *A. baumannii* results in a reservoir of resistance genes by horizontal transfer, particularly using mobile genetic elements [9]. Our findings demonstrated the emergence of pandrug-resistant *A. baumannii*, underlying the urgent need to develop alternative antibacterials with low resistance rates [10]. Increasing interest in peptidoglycan (PG) degrading enzymes (endolysins) encoded by bacteriophages has been reported due to their capacity to lyse host bacterial cell walls at the end of the lytic cycle [11,12] and their potential to treat Gram-negative bacterial infections [13,14]. A unique feature of endolysins is their modular structure, which often consists of an enzymatic activity domain (EAD) and a cell wall binding domain (CBD), allowing easy manipulation to generate novel endolysins with improved properties. Compared to bacteriophages, the use of endolysins presents promising advantages, such as a broad host range, nontoxicity, and a low probability of resistance development [15], making endolysins important candidates as alternative antibacterials. However, the outer membrane (OM) of Gram-negative bacteria represents a highly effective permeability barrier, thereby becoming insensitive to exogenously applied endolysins. Engineered endolysin versions have been released to overcome this obstacle [16]. The newly engineered endolysins called Artilysin^®^s are optimized, engineered fusions of a selected endolysin with a membrane-penetrating peptide. The latter interacts with the cation binding sites of the LPS molecules by displacing divalent cations, leading to instability and disruption of the membrane [15]. This disruption facilitates the efficient transfer of the endolysin across the OM of Gram-negative bacteria to target the peptidoglycan layer, resulting in cell wall destabilization and subsequent cell death [17].

In this study, we characterized the antimicrobial efficacy of Artilysin^®^ Art-Top3 against a panel of clinical MDR isolates of *A. baumannii*. Art-Top3 was also evaluated for its potential to kill *A. baumannii* residing inside a bacterial biofilm and in a *Galleria mellonella* infection model. The results obtained show the promising use of Art-Top3 as a novel therapeutic agent.

## 2. Results

### 2.1. Art-Top3 Displays High Antibacterial Activity and Killing Rate Against A. baumannii

To determine the activity spectrum of Art-Top3, the minimum inhibitory concentration (MIC) was tested against a panel of *A. baumannii* strains and compared with antibiotics of different classes. The antimicrobial activity of Art-Top3 showed a broad spectrum of activity against all tested strains, including multidrug-resistant isolates with an MIC of 2.5–5 µg/mL. There was no link between the antibiotic sensitivity of conventional antibiotics and Art-Top3 (Table 1).

**Figure 1:**
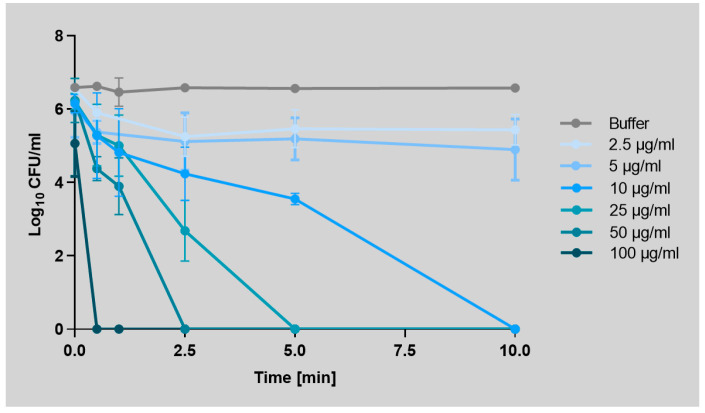
Time-dependent inhibition curve of Art-Top3 against *A. baumannii* ATCC 19606^T^. 4-(2-hydroxyethyl)-1-piperazine ethanesulfonic acid (HEPES)/NaCl solution was used as a buffer. The log_10_ colony forming unit/mL for all groups was determined at time 0 and at subsequent time points up to 10 min. Data expressed as means ± SD (n = 3). Statistical significance was determined using a two-tailed unpaired Student’s *t*-test.

The effectiveness of Art-Top3 was tested against five carbapenem-resistant *A. baumannii* patient isolates and the type strain ATCC 19606^T^. Art-Top3 was highly active against all *A. baumannii* isolates tested and resulted in a complete elimination of ≥5 log in the number of viable bacterial cells for all *A. baumannii* isolates after one hour of incubation (Figure S1). The killing ability of *A. baumannii* ATCC 19606^T^ over time was analyzed with different Art-Top3 concentrations. Application of Art-Top3 concentrations of 10–100 µg/mL led to complete killing within 10 min, whereas low concentrations (2.5–5 µg/mL) showed a lower but readily detectable effect in the period examined (Figure 1). This indicates a rapid and dose-dependent killing of Art-Top3.

### 2.2. Art-Top3 Decreases Mature A. baumannii Biofilm In Vitro

**Figure 2:**
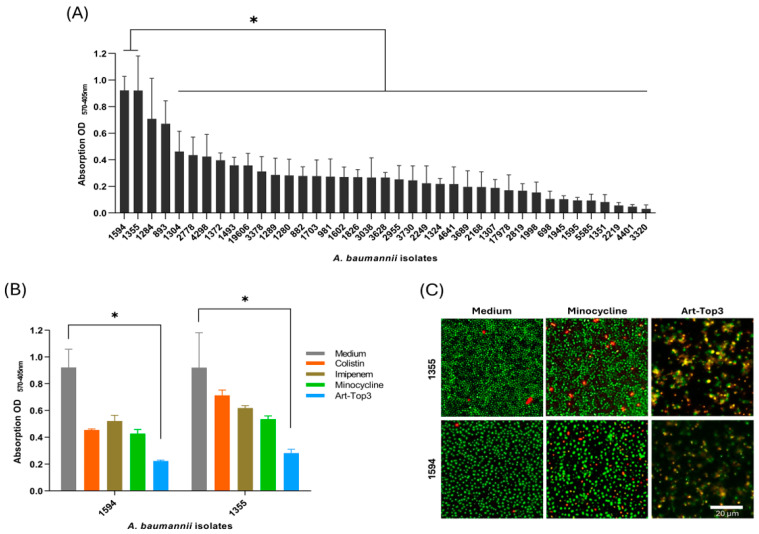
Effect of Art-Top3 on *A. baumannii* biofilms. (**A**) Biofilm formation by clinical isolates of *A. baumannii* was determined by a microtiter plate assay. Cultures were incubated overnight in microtiter plates and stained with crystal violet to quantify biofilm formation. Data are expressed as means ± SD (n = 3). (**B**) Absorption values (570–405 nm) of untreated and Art-Top3 treated biofilms. Biofilm of *A. baumannii* was grown and then treated with Mueller Hinton broth (MHB) as medium, 10xMIC Art-Top3 or 10xMIC of the antibiotics colistin, imipenem, and minocycline. The residual biofilm was assessed by crystal violet staining. Data expressed as means ± SD (n = 3). Statistical significance was determined using one-way ANOVA (* *p* < 0.05). (**C**) Representative immunofluorescence microscopic images of LIVE/DEAD stained *A. baumannii* biofilm formed and treated under dynamic conditions (scale bar: 20 μm). SYTO9 (green) staining of viable bacterial cells and propidium iodide (red) staining of dead bacterial cells.

Biofilm formation of clinical *A. baumannii* isolates was analyzed by using a microtiter-based biofilm assay to screen for strong biofilm formers. Biofilm production varied extensively among isolates, ranging from an OD_570_ of 0.03 to 0.92 (Figure 2A). *A. baumannii* isolates 1594 and 1355 showed the highest biofilm production of all isolates, and therefore, were used to evaluate the activity of Art-Top3 on *A. baumannii* biofilms (Figure 2B). The biofilm was allowed to form in a 96-well plate for 24 h before being treated with Art-Top3 or the antibiotics colistin, imipenem, and minocycline and stained with crystal violet. Art-Top3 and minocycline, a potent antibiotic against *A. baumannii* with a low resistance rate of 30.5% [18], were found to be more effective than the other antibiotics tested, with Art-Top3 showing significant biofilm reduction upon 2 h of incubation (selected time point based on Figure 1), lysing 75% of the biofilm. However, the antibiotics used did not show significant biofilm reduction, with a reduction of 40–50% in isolate 1594 and 20–40% in isolate 1355. Furthermore, the activity of Art-Top3 and minocycline was qualitatively tested under dynamic conditions and verified using LIVE/DEAD staining and immunofluorescence microscopy, which showed a blurred morphology of *A. baumannii*, indicating lysis of the cells within the biofilm (Figure 2C).

### 2.3. Art-Top3 Displays Low Toxicity Towards Human Cells

**Figure 3:**
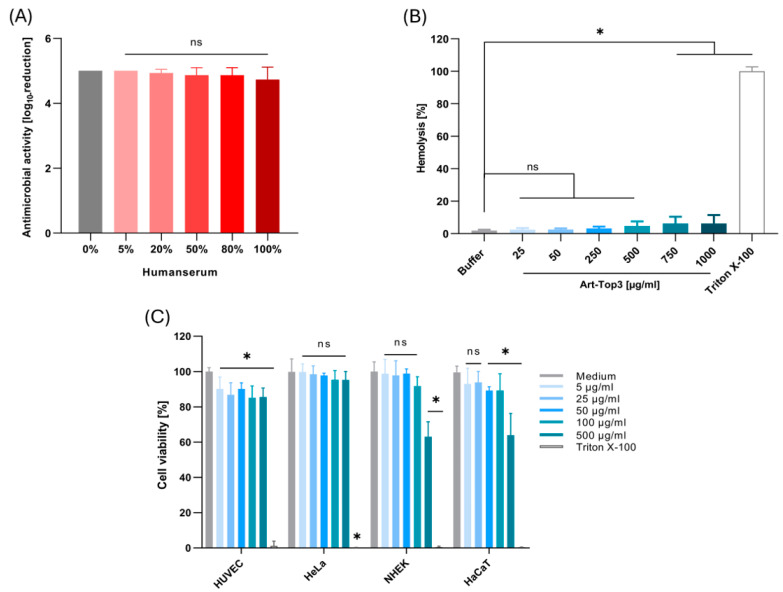
Serum activity, hemolysis, and cytotoxicity. (**A**) The antibacterial activity of 10 µg Art-Top3 against *A. baumannii* in the presence of human serum was determined by a CFU reduction assay. Data are expressed as means ± SD (n = 3). (**B**) Hemolytic activity of various Art-Top3 concentrations. Freshly prepared human red blood cells were treated with Art-Top3 at 37 °C for 24 h. Phosphate-buffered saline (PBS) was used as a buffer. Data are expressed as means ± SD (n = 3). (**C**) Viability of human cell lines after exposure to Art-Top3. The bar graph represents the percentage of viable cells compared to the medium control after exposure to different Art-Top3 concentrations. An appropriate cell culture medium (ECGM, KGM, and RPMI 1640) was used. Data are expressed as mean ± SD (n = 3). Statistical significance was determined using one-way ANOVA (ns: not significant; * *p* < 0.05).

The activity and stability of Art-Top3 were evaluated by testing the activity against *A. baumannii* in human serum. Art-Top3 (10 µg) maintained its activity in a buffer containing up to 100% serum, showing a mean bacterial reduction of 4.3 log (Figure 3A). Art-Top3 exhibited a low hemolysis of 2.5% at Art-Top3 concentrations of 25 and 50 µg/mL. Low hemolysis, such as 3.2–6.2%, also appeared at Art-Top3 concentrations of 250–1000 µg/mL in comparison to the Triton X-100 control (Figure 3B). The toxicity of Art-Top3 was also evaluated on human cell lines by using the colorimetric 3-(4,5-dimethylthiazol-2-yl)-2,5-diphenyltetrazolium bromide (MTT) assay. Human cells were incubated with various concentrations of Art-Top3, and cell viability was analyzed via an MTT kit. Art-Top3 concentrations of 5 and 50 µg/mL showed a slight decrease of 10% in human umbilical vein endothelial cells (HUVECs) viability, while 25 µg/mL, as well as higher Art-Top3 concentrations of 100 and 500 µg/mL, showed a 15% decrease in viability (Figure 3C). Human epithelial cells (HeLa-229) showed no decrease in viability even at high Art-Top3 concentrations, whereas normal human epidermal keratinocytes (NHEKs) and immortalized human keratinocytes (HaCaTs) showed a decrease in viability of approximately 35% at the highest concentration of 500 µg/mL.

### 2.4. Art-Top3 Reduces Adhesion of A. baumannii to Human Cells

**Figure 4:**
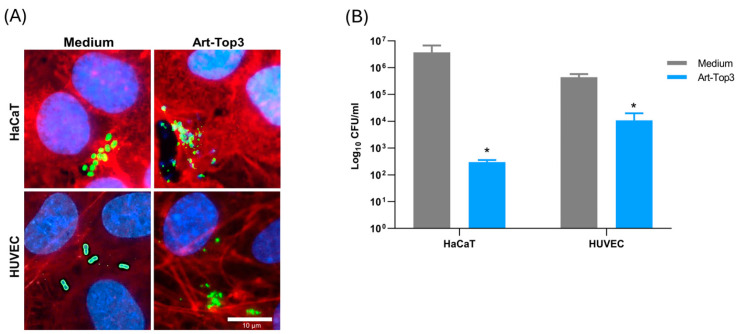
Antibacterial effectiveness of Art-Top3 on *A. baumannii* infected human cells. (**A**) Immunofluorescence microscopy of *A. baumannii* infected human cells treated with Art-Top3 or cell culture medium (ECGM and RPMI 1640). The actin cytoskeleton was stained with tetramethylrhodamine-phalloidin (red), cell nuclei with 4′,6-diamidino-2-phenylindole (DAPI, blue), and bacteria with Alexa 488 (green). Scale bar: 10 µm (**B**) Bacterial survival rate after incubation with various Art-Top3 concentrations or cell culture medium (ECGM and RPMI 1640). The number of remaining log_10_ CFU/mL was determined by plating 10-fold serial dilutions. Data are expressed as means ± SD (n = 3). Statistical significance was determined using a two-tailed unpaired Student’s *t*-test (* *p* < 0.05).

Since Art-Top3 showed low toxicity to human cells (Figure 3), its antibacterial activity at a non-toxic concentration of 50 µg/mL Art-Top3 was tested in vitro on *A. baumannii* infected HaCaT and HUVEC. Human cells were infected with *A. baumannii* as described earlier [19] and treated with medium or Art-Top3. Treatment with Art-Top3 showed a two- to three-fold reduction in bacteria. Immunofluorescence microscopy of *A. baumannii* infected HaCaT and HUVEC confirmed the effectiveness of Art-Top3, in which the bacteria appeared lysed (Figure 4A). The bacterial survival rate was determined by evaluating the CFU after therapy with Art-Top3. Without treatment, 4.3 × 10^6^ CFU/mL HaCaT and 4.4 × 10^5^ HUVEC were detected (Figure 4B). In contrast, with Art-Top3 addition, only 3 × 10^2^ CFU/mL HaCaT and 1 × 10^4^ HUVEC were detected.

### 2.5. Art-Top3 Permeabilizes the Outer Membrane and Degrades Peptidoglycan

**Figure 5:**
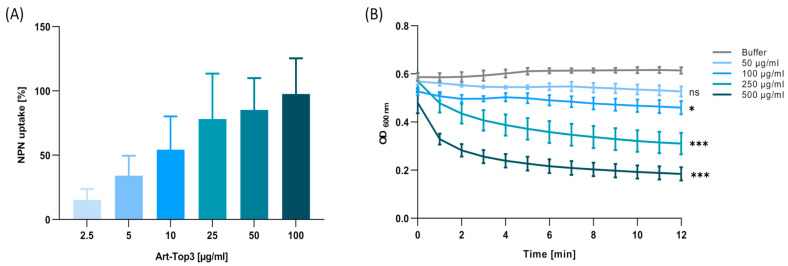
Mechanism of Art-Top3. (**A**) Outer membrane permeabilization by Art-Top3 quantitated using NPN uptake assay. The increase in fluorescence intensity was measured after 1 h at an excitation wavelength of 350 nm and an emission wavelength of 420 nm. A total of 4 µg/mL of colistin was used as positive control and 5 mM HEPES as buffer control. Data are expressed as means ± SD (n = 3). (**B**) Muralytic activities of Art-Top3 characterized by turbidity on chloroform/Tris-HCl permeabilized *A. baumannii*. HEPES/NaCl solution was used as a buffer. Data are expressed as means ± SD (n = 3). Significant differences for the final time points were determined using one-way ANOVA (ns: not significant; * *p* < 0.05; *** *p* < 0.005).

To confirm the membrane-destabilizing effect of Art-Top3, the underlying mechanisms responsible for the antibacterial activity were investigated in detail. First, the 1-N-phenylnaphthylamine (NPN) uptake assay was used to determine the OM permeability by Art-Top3. Art-Top3 increased the fluorescent intensity in ATCC 19606^T^ (Figure 5A), proving that the Art-Top3 permeabilizes the OM similarly to colistin. Next, the degradation of peptidoglycan by Art-Top3 was analyzed using a muralytic assay. A decrease in turbidity after adding ≥250 µg/mL Art-Top3 confirmed the muralytic activity of Art-Top3 (Figure 5B).

### 2.6. Art-Top3 Ameliorates Survival of Galleria mellonella Larvae After Infection with Multidrug-Resistant A. baumannii

**Figure 6:**
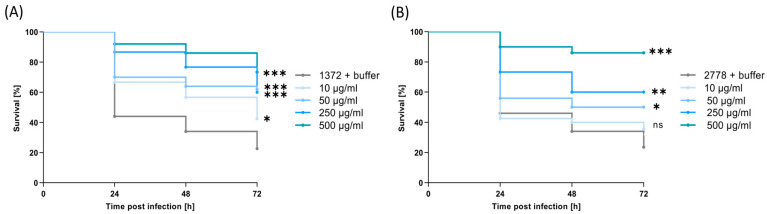
In vivo activity of Art-Top3 in the *G. mellonella* infection model. (**A**) Survival curves for *G. mellonella* infected with 10^6^ to 10^8^ CFU of *A. baumannii* isolate 1372 and (**B**) *A. baumannii* isolate 2778 followed by injection with HEPES/NaCl buffer solution (buffer group) or 10–500 μg/mL Art-Top3 (treatment group) (n = 5). Statistical significance was determined using a two-tailed unpaired Student’s *t*-test (ns: not significant; * *p* < 0.05; ** *p* < 0.01; *** *p* < 0.005).

With the confirmation of Art-Top3’s antibacterial effectiveness in vitro, its efficacy in vivo was examined using larvae of the greater wax moth (*Galleria mellonella*). *G. mellonella* were infected with the multidrug-resistant *A. baumannii* patient isolates 1372 and 2778 and subsequently injected with Art-Top3 or buffer as a control. These patient isolates were included in the experiments because they belong to the sequence type ST2 and to the international cluster IC2, the most abundant clonal lineage worldwide [20]. In the two control groups without Art-Top3 treatment, approximately 55% of the *G. mellonella* larvae died 24 h post infection (Figure 6A,B). The mortality rate rose to 78% after 72 h. In contrast, the Art-Top3 treated groups were concentration-dependent, as a high Art-Top3 concentration also resulted in dramatically higher survival rates. Accordingly, using 250–500 µg/mL Art-Top3 leads to the survival of 87–92% *G. mellonella* 24 h after injection. After 72 h, the survival rate decreased at an average of 60–74% compared to 23% in the control group, indicating that Art-Top3 kills multidrug-resistant *A. baumannii* in vivo (Figure 6A,B).

## 3. Discussion

Endolysins are promising antibacterials with high specificity that can support or even replace antibiotics [21]. The antibacterial activity of engineered endolysins depends on the biochemical properties of the endolysin and the target bacteria, as each bacterium has differences in peptidoglycan composition and structure [22]. Here, we used a new class of engineered endolysin, termed Artilysin^®^ Art-Top3, with an outer membrane-penetrating peptide that exhibited activity against clinical multi-resistant isolates of *A. baumannii* (Table 1). It has been demonstrated that Art-Top3 showed a faster (30 s) killing of *A. baumannii* (Figure 1) than other engineered endolysins in previously described studies (5–15 min), albeit the comparison of different endolysins and experimental settings is difficult [15,17].

Bacterial biofilms are a survival strategy to resist the host defense mechanism or sub-optimal environmental conditions [23]. Art-Top3 was not only effective against numerous clinical *A. baumannii* isolates in vitro but also significantly killed bacteria in biofilms under static and dynamic conditions (Figure 2). *A. baumannii*, being an opportunistic pathogen, is known for its improved survival capability through biofilm formation and persistence in hospital environments [24]. Art-Top3’s significant ability in killing biofilm-associated bacteria could be harnessed by disinfecting medical devices, like ventilators or catheters, when applied topically [25]. Furthermore, Art-Top3 could be applied topically to skin and wounds to prevent the development of wound infections, associated with biofilm formation, or as a treatment option when an infection is already present [26]. A possible future medical application might be a therapeutic spray formulation used for topical treatment of, e.g., infected wounds.

The stability and activity of Art-Top3 were analyzed in human serum to determine the potential clinical use in wound infections. Art-Top3 was able to maintain its 99.9% activity in up to 100% serum (Figure 3A). The lower activity of endolysins in serum has been reported previously (down to 4–10%; [17,27]), possibly caused by the interaction of differentially charged peptides (e.g., albumin). However, the stability and activity of Art-Top3 should be further tested in wound fluids, considering that factors like proteases and pH can significantly influence lytic activity.

Endolysins have been identified as potential triggers of the immune system as they represent a foreign protein [28]. In consequence, this could lead to premature degradation of endolysins and to undesirable inflammatory reactions in the human body [29]. Therefore, endolysins are mainly used topically in humans, i.e., externally on the skin or mucous membranes [30]. We examined the toxicity of Art-Top3 on human red blood cells and human cell lines for clinical use on skin and wounds. Even at high concentrations 500–1000 µg/mL, Art-Top3 showed low toxic effects of 4.7–6.2% on human red blood cells. Similarly, low hemolytic activity (<14%) has been shown in previously published studies [31,32]. Moreover, Art-Top3 displayed cytotoxicity of 5–15% in human cell lines (HUVEC, HeLa-229; Figure 3B,C). In contrast, both keratinocyte cell lines NHEK and HaCaT showed diminished viabilities of ~65% upon treatment with 500 µg/mL Art-Top3 compared to untreated controls (Figure 3C). Previous studies showed that endolysins exhibit little or no toxicity to immortalized cell lines, like HeLa-299, HaCaT, and A549, even at concentrations of up to 200 µM [31,33,34]. This indicates that, especially, primary skin cells like NHEK might be more susceptible to toxic effects at very high concentrations of Art-Top3. Furthermore, this suggests that Art-Top3 might be used in future applications in vivo, such as clinical trials and animal experiments, at lower concentrations (e.g., 50 µg/mL) to avoid impaired cell viability and, therefore, wound healing.

We aimed to decipher the mechanism responsible for the antibacterial activity of Art-Top3. First, the NPN-uptake was used to determine the OM permeability. The OM permeabilization of Art-Top3 was similar to colistin (Figure 5A), indicating an interaction of the fused peptide with negatively charged OM, making *A. baumannii* permeable to Art-Top3 and increasing access of the lysin to the peptidoglycan layer. Second, peptidoglycan degradation by Art-Top3 was observed in the muralytic assay (Figure 5B), indicating that the muralytic activity of Art-Top3 was from cleavage of the bonds and a loosening of the PG architecture. These results are consistent with the OM permeabilization and muralytic activity of previously described lysins, such as LysAB2 [17].

The *G. mellonella* infection model was employed to analyze the activity of Art-Top3 against *A. baumannii* infections in vivo. *G. mellonella* larvae have been shown to be suitable hosts for analyzing the virulence of *A. baumannii* and the impact of antibacterials during infection [35,36]. Art-Top3 could significantly rescue larvae in a dose-dependent manner by injecting Art-Top3 after infection with multidrug-resistant *A. baumannii* patient isolates (Figure 6), suggesting that Art-Top3 has significant antimicrobial activity in vivo. The survival rate of *G. mellonella* in our experiment of ≥87% after 24 h was similar to or even higher than the survival rates of 60% and ≥80%, respectively, by other previously published studies [17,37]. However, the in vivo activity of Art-Top3 should be confirmed in a mammalian model (e.g., mice), considering that in the *G. mellonella* model, the lack of standardized procedures, especially in larval culture, storage, and injection, limits the study.

Our study proves that Art-Top3 can be used to combat infections caused by *A. baumannii* with high efficacy and stability. The unique rapid efficacy of Art-Top3 could be beneficial in the treatment of acute infections. Art-Top3 demonstrated high activity in various experimental setups and showed potential for a wide range of applications ranging from various medical applications to disinfection, veterinary, food, feed, and agricultural applications. Taken together, Art-Top3 exerts high bacterial activity against clinical MDR *A. baumannii* strains in vitro and in vivo with low toxicity to primary human cells in vitro. In contrast to other endolysins, Art-Top3 shows a very fast onset of action and high stability in serum. In future studies, the development of resistances against Art-Top3 should be analyzed to avoid a rapid and widespread resistance to novel antibacterials.

## 4. Materials and Methods

### 4.1. Engineering of Artilysin^®^

Artilysin^®^s are a registered trademark by Lysando AG (Triesenberg, Lichtenstein). Artilysin^®^ Art-Top3 was engineered by fusing an endolysin to an OM destabilizing peptide that is active against Gram-negative bacteria. The prophage endolysin encoded in the *A. baumannii* AB5075 genome was engineered for improved expression and stability [38]. The fused OM destabilizing peptide belongs to the cathelicidin family of antimicrobial peptides (CAMPs). The DNA sequence encoding the peptide and the engineered endolysin was cloned between the NdeI and XhoI restriction sites in the multiple cloning site of the pET21b expression vector (Novagen, Darmstadt, Germany). The final expression construct also contained an enterokinase protease-cleavable N-terminal 6xHN affinity tag for IMAC purification.

Recombinant expression of Art-Top3 was performed in *Escherichia coli* BL21(DE3). The *E. coli* cells were grown in an autoinduction medium at 37 °C for 4 h, then the temperature was reduced to 16 °C, and protein expression was continued overnight. The *E. coli* cells were harvested by centrifugation for 15 min at 4000× *g* and 4 °C. The cell pellet was resuspended in buffer (20 mM HEPES, 1 M NaCl, 10 mM imidazole) containing 0.05 mg/mL DNase I (Applichem, Darmstadt, Germany) and protease inhibitor (cOmplete, EDTA-free Protease Inhibitor Cocktail, Roche, Mannheim, Germany). Cells were disrupted via sonication on ice. Cell debris was separated by centrifugation for 30 min at 13,000× *g* and 4 °C. The supernatant was loaded on a 5 mL HisTrap FF column (GE Healthcare, Frankfurt, Germany) and Art-Top3 was purified by Ni^2+^ affinity chromatography (Äkta FPLC, GE Healthcare, Frankfurt, Germany) using the N-terminal 6xHN-tag. The elution from the column was performed using a linear gradient to 100% IMAC elution buffer (20 mM HEPES, 500 mM NaCl, 500 mM imidazole, pH 7.4). The elution fractions containing Art-Top3 were pooled and diluted to a final NaCl concentration of 200 mM and further purified using ion-exchange chromatography (combination of HiTrap Capto Adhere and HiTrap SP FF, GE Healthcare, Frankfurt, Germany). The elution from the column was performed using a linear gradient to 100% IEX elution buffer (20 mM HEPES, 1 M NaCl, pH 7.4). The fractions containing Art-Top3 were pooled, diluted to 200 mM NaCl, and incubated with enterokinase protease (New England Biolabs, Frankfurt, Germany) for two days at room temperature to cleave the N-terminal affinity tag. In a final step, Art-Top3 was separated from the enterokinase and the cleaved affinity tag by a second ion-exchange chromatography (combination of HiTrap Capto Adhere and HiTrap SP FF, GE Healthcare, Frankfurt, Germany). The elution from the column was performed using a linear gradient to 100% IEX elution buffer (20 mM HEPES, 1 M NaCl, pH 7.4). The fractions containing Art-Top3 were pooled and dialyzed against the final protein buffer (20 mM HEPES, 150 mM NaCl, pH 7.4). The purified Art-Top3 was adjusted to a concentration of 6.28 mg/mL in 20 mM HEPES and 150 mM NaCl (pH 7.4) and stored at 4 °C.

### 4.2. Bacterial Strains and Culture Conditions

Clinical isolates of *A. baumannii* were recovered from patients hospitalized at the Goethe University Hospital in Frankfurt. Reference strain *A. baumannii* ATCC 19606^T^ [39] was obtained from the German Collection of Microorganisms and Cell Cultures (DSMZ, Braunschweig, Germany). All isolates were cultured on Columbia blood agar (CBA) plates (Thermo Scientific™ Oxoid™, Darmstadt, Germany) at 37 °C.

### 4.3. Antimicrobial Susceptibility Testing

The bacterial strains used are described in the Supplemental Material (Table S1). Antimicrobial susceptibility was determined by antibiotic gradient strips (Liofilchem^®^ MIC Test Strip, Liofilchem, Roseto degli Abruzzi, Italy) and Mueller Hinton Agar (Oxoid, Wesel, Germany). Colistin sulfate (Sigma-Aldrich, Darmstadt, Germany) susceptibility was determined by broth microdilution using cation-adjusted Mueller Hinton broth (CAMHB; Becton Dickinson Bioscience, Heidelberg, Germany) and increasing concentrations (0–256 µg/mL). Minimum inhibitory concentrations were evaluated according to EUCAST (v 13.1) and CLSI (33rd Edition) guidelines. The susceptibility of Art-Top3 was tested by broth microdilution in Mueller Hinton broth (MHB; Becton Dickinson Bioscience, Heidelberg, Germany). An overnight culture was diluted 1:5000 to a concentration of 10^5^ CFU/mL in MHB. Art-Top3 was added at different concentrations (0–30 µg/mL). The mixtures were incubated overnight (18 h) at 37 °C. MHB with bacteria and MHB with 20 μL of HEPES/NaCl (Sigma-Aldrich, Hamburg, Germany; Fresenius, Bad Homburg, Germany) solution were included as positive and negative controls. Bacterial growth was determined by turbidity measurements.

### 4.4. Antibacterial Activity Assay

Logarithmic phase cultures of *A. baumannii* were prepared by inoculating MHB with an overnight culture at 37 °C to OD_600_ of 0.6. Then, bacteria were harvested by centrifugation (16,000× *g* for 5 min) and 10-fold diluted in 20 mM HEPES (pH 7.4). Bacterial dilution was incubated with 10 µg of Art-Top3 in 20 mM HEPES and 150 mM NaCl (pH 7.4) in a final volume of 100 µL at 37 °C, with shaking for 60 min. Thereafter, treated bacteria were diluted and plated on CBA plates. For the time-killing assay, 1–100 µg/mL of Art-Top3 was incubated with the log-phase bacteria. After 0.5–10 min of incubation at 37 °C, samples were taken, diluted, and plated on CBA plates. HEPES/NaCl solution with bacteria was used as the positive control.

### 4.5. Evaluation of Biofilm Formation

*A. baumannii* strains were grown in tryptic soy broth (TSB; Becton Dickinson Bioscience, Heidelberg, Germany) without dextrose overnight at 37 °C, with shaking. For the static biofilm assay, the overnight culture was diluted to OD_600_ of 0.5, transferred into a 96-well plate Nunclon Delta-Treated (Thermo Scientific™ Nunc™, Darmstadt, Germany), and incubated overnight at 37 °C. Plates were washed three times with phosphate-buffered saline (PBS; Gibco, Karlsruhe, Germany), treated with 10xMIC Art-Top3 or the antibiotics colistin, imipenem (Thermo Fisher Scientific, Dreieich, Germany), and minocycline (Sigma-Aldrich, Darmstadt, Germany) or MHB as the positive control and incubated at 37 °C for 2 h. Treated biofilm was washed, all media were removed, and the biofilm fixed at 65 °C for 2 min. After fixation, the biofilm was stained with crystal violet (0.1%; Roth, Karlsruhe, Germany) for 2 min. Then, the biofilm was washed with distilled water and dissolved by adding ethanol absolute (Sigma-Aldrich, Darmstadt, Germany). To quantify the crystal violet staining, the optical density at 570 nm was measured using a microplate reader (infinite M200 PRO, Tecan, Männedorf, Switzerland).

For evaluation of biofilm formation under dynamic conditions, a diluted overnight culture was used to inoculate a six-channel μ-Slide (ibidi GmbH, Martinsried, Germany) with 100 µL per channel. After an incubation of 1 h at 37 °C, sterile 10 mL syringes containing MHB were connected to a syringe pump (PHD Ultra, Havard Apparatus, Cambridge, MA, USA). A constant flow rate of 1 µL/min was maintained for 24 h. To remove planktonic cells, MHB was flushed for 60 min, followed by Art-Top3 or MHB as the positive control for 24 h. Then, the biofilm was washed three times and stained with 5 µM SYTO9 (Invitrogen, Darmstadt, Germany) and 30 µM propidium iodide (Becton Dickinson Bioscience, Heidelberg, Germany) for 15 min to distinguish between live and dead cells. Biofilms were analyzed using a Zeiss Axio Imager 2 microscope equipped with a Spot RT3 microscope camera (Diagnostic Instruments Inc., Sterling Heights, MI, USA) and operated by VisiView V.2.0.5 (Visitron Systems, Puchheim, Germany).

### 4.6. Biocompatibility Assay

Healthy human blood was collected and centrifuged at 2000 rpm for 5 min. Human red blood cells (RBCs) were washed with PBS. An 8% (*v*/*v*) RBC suspension was prepared in a 96-well plate (Greiner, Frickenhausen) and mixed with different concentrations of Art-Top3. PBS and Triton X-100 (0.1%; Sigma-Aldrich, Darmstadt, Germany) were used as a negative and a positive control. After an incubation of 1 h at 37 °C, the 96-well plate was centrifuged at 1000 rpm for 5 min at 4 °C. Supernatants from each well were transferred to a new sterile 96-well plate and hemolysis was evaluated by measuring the optical density at 405 nm using a microplate reader (infinite M200 PRO, Tecan). All experiments were performed in triplicate.

HUVEC, NHEK, HaCaT, and HeLa-229 were used to measure the toxicity of Art-Top3 by MTT assay (Roche, Mannheim, Germany). The cells were seeded at a density of 10^5^ cells/well in a 96-well plate for 24 h. Next, 5–500 µg/mL Art-Top3 was added and incubated for 24 h at 37 °C. Cell culture medium and Triton X-100 (0.1%) were used as a negative and positive control, respectively. The treated cell culture was incubated with MTT (0.5 mg/mL) for 4 h at 37 °C. Subsequently, the purple formazan crystals were solubilized by adding a solubilization solution (Roche, Mannheim, Germany). The optical density was measured after 24 h at 570 nm using a microplate reader (infinite M200 PRO, Tecan).

### 4.7. Cell Culture and Infection

HUVECs were cultivated using endothelial cell growth medium with supplement mix (PromoCell, Heidelberg, Germany) and 10% fetal calf serum (Sigma-Aldrich, Darmstadt, Germany). NHEKs were cultivated using keratinocyte growth medium 2 with a supplement mix (PromoCell). HaCaT and HeLa-229 were cultivated using RPMI 1640 (PAN Biotech, Aidenbach, Germany). Cell lines were incubated in a 5% CO_2_ atmosphere at 37 °C and a humidity of 95%.

For infection experiments, HUVECs and HaCaT were seeded at a density of 2–5 × 10^5^ cells per well. Bacterial cultures were adjusted to an OD_600_ of 0.2. Thereafter, bacteria were centrifuged (3000× *g* for 10 min) and washed with PBS. Bacteria were resuspended in the appropriate cell culture medium and adjusted to the required multiplicity of infection (MOI 100). After 3 h of infection, cells were treated with Art-Top3 or cell culture medium for 1 h at 37 °C. The number of viable *A. baumannii* was quantified from serial dilutions on CBA plates and subsequent counting of CFU units. For staining and immunofluorescence microscopy of HaCaT and HUVEC, cells were fixed using 3.75% paraformaldehyde (PFA; Thermo Fisher Scientific, Dreieich, Germany) for 10 min at 4 °C and permeabilized with 0.2% Triton X-100 for 15 min. After blocking with 1% bovine serum albumin for 1 h, cells were incubated at room temperature for 1 h using the primary antibody rabbit anti-*A. baumannii* [40] and subsequently with secondary IgG-antibody Alexa 488 conjugated anti-rabbit IgG (Dianova, Hamburg, Germany). Actin cytoskeleton was stained with TRITC Phalloidin (Sigma-Aldrich, Darmstadt, Germany). Bacterial and mammalian DNA were stained with DAPI (Merck, Darmstadt, Germany) for 10 min. Coverslips were mounted using a fluorescence mounting medium (S3023, Dako, Hamburg, Germany).

### 4.8. Human Serum Assay

For evaluation of the antibacterial activity of Art-Top3 in human serum, log phase cultures of *A. baumannii* were washed once and resuspended in 20 mM HEPES. Then, bacteria (approximately 10^6^ CFU/mL) were treated with 100 µg/mL Art-Top3 in the presence of 0−100% human serum at 37 °C for 60 min. PBS was used as the positive control. The viable cell numbers were enumerated by plating on CBA plates.

### 4.9. Outer Membrane Permeability Assay

The OM permeability of Art-Top3 was examined by the NPN uptake assay [41]. Using the neutral hydrophobic fluorescent probe 1-N-phenylnaphthylamine (NPN; Thermo Fisher Scientific, Dreieich, Germany), the destabilization of OM can be measured by the fluorescence signal enhancement due to the enrichment of NPN in the hydrophobic membrane. *A. baumannii* was grown to the mid-log phase, centrifuged, and resuspended in PBS. Then, the bacterial suspension was incubated with NPN to a final concentration of 10 μM and varying concentrations (2.5−100 μg/mL) of Art-Top3, 4 µg/mL colistin, or 5 mM HEPES for 1 h. The fluorescence intensities were recorded using a microplate reader (infinite M200 PRO, Tecan, Männedorf, Switzerland) at 350 nm for excitation and 420 nm for emission. All experiments were performed in triplicate. The NPN uptake of Art-Top3 was calculated by subtracting the fluorescence of NPN with *A. baumannii* and Art-Top3 from the fluorescence of NPN with *A. baumannii* divided by the subtraction of the fluorescence of NPN with *A. baumannii* and Art-Top3 from the fluorescence of NPN with *A. baumannii* and colistin.

### 4.10. Muralytic Assay

*A. baumannii* ATCC 19606^T^ (CFU: 1 × 10^8^/mL corresponding to an OD_600_ of 0.5) was treated with chloroform/buffer to permeabilize the OM as described by Chen et al., 2021 [17]. Art-Top3 concentrations from 50 to 500 μg/mL were used and absorbance was measured at 600 nm using a microplate reader (infinite M200 PRO, Tecan). HEPES/NaCl solution was used as the positive control.

### 4.11. Galleria Mellonella Infection Model

The larvae of the greater wax moth (*G. mellonella*) infection model were used as described by [6] to assess the antibacterial effectiveness of Art-Top3 in vivo. Larvae were acquired from Sud Est Appats (Queige, France). The larvae were infected by injecting bacterial suspensions corresponding to 10^6^ to 10^8^ CFU into the last left proleg using a Hamilton precision syringe. Then, 10–500 µg/mL of Art-Top3 (treatment group) or 10 µL of PBS solution (control group) was injected into the last right proleg within 30 min. Treated larvae were incubated at 37 °C and survival was monitored for 72 h.

### 4.12. Data Analysis and Statistics

All statistical analyses were performed using GraphPad Prism V7 (GraphPad, San Diego, CA, USA). The Student’s t-test and one-way ANOVA were used to compare the differences between the treated and untreated bacterial cultures. The log-rank test (Mantel–Cox test) was used to analyze Kaplan−Meier survival analysis. Statistical significance was defined as *p* < 0.05. The number of replicates is indicated in each figure.

## Figures and Tables

**Table 1:** MICs of Art-Top3 against different *A. baumannii* patient isolates and the type strain ATCC 19606^T^.

		MIC [µg/mL]							
Strain	Resistance	Art-Top3	Piperacillin-Tazobactam	Ceftazidime	Imipenem	Ciprofloxacin	Amikacin	Minocycline	Colistin
1355	MDR	2.5	≥32	≥32	≥32	4	≥32	0.125	0.25
1594	MDR0	2.5	≥32	≥32	≥32	≥32	≥32	0.125	1
ATCC 19606^T^	WT	5	24	12	1	0.25	16	0.125	0.5
698	MDR	5	≥32	≥32	≥32	≥32	≥32	0.064	1
893	MDR	5	≥32	≥32	≥32	≥32	≥32	0.5	1
981	MDR	5	≥32	≥32	≥32	≥32	≥32	0.5	0.25
1284	MDR	5	≥32	≥32	≥32	≥32	≥32	1	1
1372	MDR	5	≥32	≥32	≥32	≥32	≥32	0.125	0.5
2778	MDR	5	≥32	≥32	≥32	≥32	≥32	16	2
3378	MDR	5	≥32	≥32	≥32	≥32	≥32	16	0.125
6863	MDR	5	≥32	≥32	≥32	≥32	≥32	0.25	0.5
6904	MDR	5	≥32	≥32	≥32	≥32	≥32	0.75	0.125

## Data Availability

The original contributions presented in this study are included in the article/supplementary material. Further inquiries can be directed to the corresponding authors.

## Supplementary Materials

The following supporting information can be downloaded at: https://www.mdpi.com/article/10.3390/antibiotics14020162/s1; Table S1: Strains used in this study to test antibacterial activity of Art-Top3. Figure S1: Antibacterial activity of 10 µg Art-Top3 against *A. baumannii* isolates after 1 h of incubation. The bacterial reduction is expressed in log_10_ units by 10-fold serial dilution and compared with the HEPES/NaCl solution-treated control.
