# Supplementary material for: Bactericidal Effect of a Novel Phage Endolysin Targeting Multi-Drug-Resistant Acinetobacter baumannii
Source: Antibiotics (Basel). 2025 Feb 6;14(2):162. doi: 10.3390/antibiotics14020162 (PMC11851708; doi:10.3390/antibiotics14020162)
Supplement: Supplementary file 1 [file antibiotics-14-00162-s001.zip › antibiotics-3412718-supplementary.pdf]

Supplementary Material

Bactericidal effect of a novel phage endolysin targeting multi-drug-resistant *Acinetobacter baumannii*

Sara Garcia Torres[1], Dirk Henrich[2], Rene D. Verboket[2], Ingo Marzi[2], Gernot Hahne[3], Volkhard A. J. Kempf[1,*] and Stephan Göttig[1,*]

[1] Goethe University Frankfurt, University Hospital, Institute of Medical Microbiology and Infection Control, Frankfurt am Main, Germany
[2] Goethe University Frankfurt, University Hospital, Department of Trauma Surgery and Orthopedics , Frankfurt am Main, Germany
[3] Lysando Innovations Lab GmbH, Regensburg, Germany
* Correspondence: goettig@med.uni-frankfurt.de

Table S1: Strains used in this study to test antibacterial activity of Art-Top3.

Strain	Specimen	ST[a]	IC[b]	Year of Isolation	Reference
1355	Wound	85	Sporadic	2011	This study
1594	Wound	85	Sporadic	2012	This study
ATCC 19606[T]	Urine	52	Sporadic	1948	[39]
698	Skin	25	7	2007	This study
893	Wound	1	1	2009	This study
981	Wound	15	4	2010	This study
1284	Wound	1	1	2011	This study
1372	Nose swab	2	2	2011	[36]
2778	Rectal swab	2	2	2015	
3378	Rectal swab	604	2	2016	This study
6863	Rectal swab	78	6	2022	This study
6904	Wound	1	1	2022	This study

[a]ST, sequence type
[b]IC, international cluster

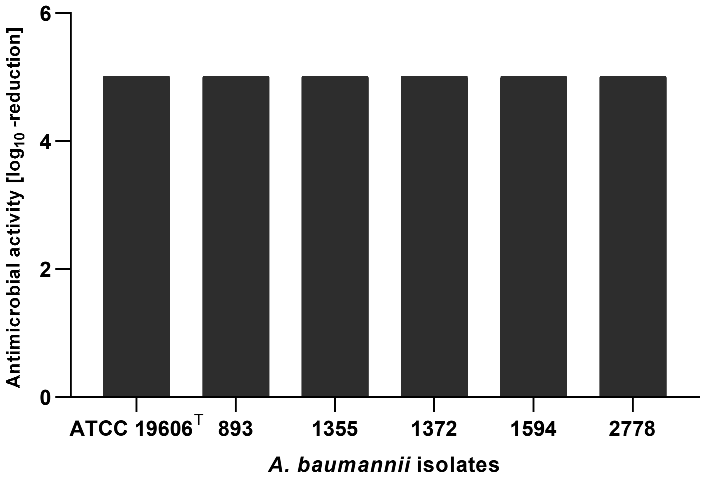

Figure S1. Antibacterial activity of 10 µg Art-Top3 against *A. baumannii* isolates after 1 h of incubation. The bacterial reduction is expressed in log_{10} units by 10-fold serial dilution and compared with the HEPES/NaCl solution-treated control.
